# Investigating Gender Differences under Time Pressure in Financial Risk Taking

**DOI:** 10.3389/fnbeh.2017.00246

**Published:** 2017-12-15

**Authors:** Zhixin Xie, Lionel Page, Ben Hardy

**Affiliations:** ^1^Queensland Behavioral Economics Group, School of Economics and Finance, Queensland University of Technology, Brisbane, QLD, Australia; ^2^School of Finance and Management, SOAS University of London, London, United Kingdom

**Keywords:** testosterone, 2D:4D, fWHR, time pressure, risk taking

## Abstract

There is a significant gender imbalance on financial trading floors. This motivated us to investigate gender differences in financial risk taking under pressure. We used a well-established approach from behavior economics to analyze a series of risky monetary choices by male and female participants with and without time pressure. We also used second to fourth digit ratio (2D:4D) and face width-to-height ratio (fWHR) as correlates of pre-natal exposure to testosterone. We constructed a structural model and estimated the participants' risk attitudes and probability perceptions via maximum likelihood estimation under both expected utility (EU) and rank-dependent utility (RDU) models. In line with existing research, we found that male participants are less risk averse and that the gender gap in risk attitudes increases under moderate time pressure. We found that female participants with lower 2D:4D ratios and higher fWHR are less risk averse in RDU estimates. Males with lower 2D:4D ratios were less risk averse in EU estimations, but more risk averse using RDU estimates. We also observe that men whose ratios indicate a greater prenatal exposure to testosterone exhibit a greater optimism and overestimation of small probabilities of success.

## Introduction

Why are there so few women trading in the markets? The last 50 years have seen more and more women participating in the workforce. In many professions, the percentage of women approaches or exceeds 50% (see for example, Chambers Partners, 2014; Kaiser Family Foundation, 2015; Catalyst, 2016). Yet some professions stay firmly outside of this evolution. Professional traders on financial trading floors are unambiguously one of these cases. Although women represent more than half the workforce in financial services (Sethi et al., 2013) they are typically in marketing, compliance or HR roles (Jäkel and Moynihan, 2016). What scant data there is suggests that women comprise 15% of junior investment and trading roles (Green et al., 2009; Lietz, 2012).

The causes of this gender imbalance are still not well understood. While in some professions it is argued that an invisible ceiling prevents the access of women, (Korzec, 2000; Williams and Richardson, 2010; Truss, 2016) this is unlikely to be the case in finance, where performance pressure pushes firms to look for the best talent at all costs. A number of explanations have been advanced in both the academic and practitioner literature for the relative absence of women. Some explanations suggest that there are fundamental differences in cognition between the sexes (e.g., Sapienza et al., 2009), some that there are psychological differences (see Charness and Rustichini, 2011) and some that social factors account for differences in behavior (Byrnes et al., 1999; Saqib and Chan, 2015) and that this, in turn accounts for the differences in representation. This study investigates a potential factor driving gender imbalance on trading floors: differences between men's and women's risk preferences, particularly under time pressure.

Trading is a pressurized activity where stakes are high and time is short (Oberlechner and Nimgade, 2005; Kocher and Sutter, 2006). To examine the relationship between risk-taking, time pressure and gender, we use a standard risk elicitation experiment with substantial incentives, where biological markers of prenatal exposure to testosterone are measured for men and women and where choices are observed under different degrees of time pressure.

This paper contributes to three distinct bodies of research: the literature on gender differences in risk attitudes, the literature on gender differences in financial behavior and careers, and the literature on stability of preferences.

There is a substantial body of research on gender differences in risk attitudes. One of the most common and consistent findings in the risk preference literature has been that men take more risk than women (Powell and Ansic, 1997; Byrnes et al., 1999; Eckel and Grossman, 2002; Croson and Gneezy, 2009). Croson and Gneezy (2009) discussed some explanations of the gender difference in risk taking, which included emotions, overconfidence and risk as challenge or threats. The search for the roots of these gender differences has pointed to the role played by the androgen hormone testosterone. Testosterone (T) is an androgenic hormone which plays a pivotal role in sexual differentiation. This organizing role of testosterone is what alters the course of fetal development from the default female pattern—in effect, it is what makes men men. In addition to this organizing and differentiating role, testosterone, is also thought to modulate behavior in a number of ways. Testosterone levels have been positively associated with a number of behaviors in adult men, including aggression (Archer, 2006), sensation seeking (Roberti, 2004), hostility (Hartgens and Kuipers, 2004), mate-seeking (Roney et al., 2003), and dominance (Mazur and Booth, 1998). Research in economics has shown that markers of pre-natal exposure to testosterone—in effect, measures of testosterone's organizing effects-have an impact on risk attitude (Coates and Page, 2009; Brañas-Garza and Rustichini, 2011; Garbarino et al., 2011; Brañas-Garza et al., 2017). We complement this research by investigating how prenatal testosterone exposure affects risk attitude decomposed into outcome sensitivity and probability sensitivity (in a RDU model).

This paper also contributes to the substantial literature on gender differences in financial behavior, which have been observed in both real and experimental markets. In the real market, men believe they are more competent than women (Graham et al., 2009), are more overconfident (Grinblatt and Keloharju, 2009), and trade more often than women (Barber and Odean, 2001). Deaves et al. (2010) found no gender effect in trading but observed that women traded less than men. Experimental studies, such as Fellner and Maciejovsky (2007), find that women submitted fewer offers and engaged in fewer trades than men. Eckel and Füllbrunn (2015) showed that all-male markets yield significant price bubbles while all-female markets produced prices that were below fundamental value. A variety of reasons have been suggested for these differences in observed behavior. Research has suggested that men are more competitive (Niederle and Vesterlund, 2007), so drive harder to beat others. Men are perceived as selfish (Aguiar et al., 2009; Brañas-Garza et al., 2016) and actually are more selfish (Rand, 2016).

One of the differences between men and women is in levels of testosterone. Coates et al. (2010) proposed a hypothesis suggesting that the irrational exuberance observed during market bubbles is mediated by testosterone. They speculated that men and women traders are likely to behave differently with male traders' behavior driving market instability. In the present study, we compare men and women's financial risk taking under time pressure. Time pressure is a key aspect of financial decisions on the trading floor. Traders make decisions in financial markets within seconds after new information becomes available (Busse and Green, 2002). In the light of this we theorized that gender differences under time pressure may be one of the factors driving the gender imbalance observed in these environments. If men and women make different decisions under time pressure then it may be that the market favors one decision making profile over another, and so favors one gender over another. Kocher et al. (2013) found that risk aversion for gains was robust under time pressure, whereas risk-seeking for losses turned into risk aversion under time pressure. For mixed prospects, i.e., a mixture of gains and losses, subjects became more loss-averse and more gain-seeking under time pressure. Nursimulu and Bossaerts (2014) found that the time-varying sensitivities translated into decreased risk aversion and increased probability distortions for gains under extreme time pressure. Capraro et al. (2017) examined the effect of time pressure and degree of deliberation on decisions about the allocation of resources. They did not, however, examine gender effects. Although there has been work on social preferences and time pressure, there is less work on risk attitudes under time pressure and very little on gender difference in risk attitudes under time pressure.

Finally, by investigating variations in risk preferences under time pressure, the paper contributes to the literature on the stability of economic preferences. The stability of preferences has been a shibboleth of much economic theory since Stigler and Becker's seminal paper (Stigler and Becker, 1977). Recent research, however, has shown that preferences are not as stable as hitherto supposed. Both explicit factors, for example time pressure (Kocher and Sutter, 2006), and implicit ones, such as levels of the hormone cortisol (Kandasamy et al., 2014), mean that people make different choices. Research in a number of fields has shown that time pressure affects the nature of interpersonal interaction, such as the levels of cooperation (Rand et al., 2012, 2014; Capraro and Cococcioni, 2015, 2016; Rand, 2016). Despite this, the impact of time pressure has been largely ignored by economics (Kocher and Sutter, 2006; De Paola and Gioia, 2016) and, what work there has been, has not clearly delineated the influence of time pressure on decision-making. Work rooted in experimental psychology has examined the speed vs. accuracy trade-off. Speedy decisions are thought to be of poorer quality, as time pressure prevents effective information processing. This, in turn, leads individuals to fall back on heuristics rather than the information presented (see Kocher and Sutter, 2006). Where risk appetite is evaluated, most research has suggested that risk-taking increases with time pressure (Huber and Kunz, 2007; Young et al., 2012; Kocher et al., 2013; Hu et al., 2015). Only Young et al. (2012) examined gender differences, but found none.

Our research finds that, in line with previous research, male participants took more risk. In addition, we identified three patterns which shed new light on gender differences in risk attitudes. First, the degree of testosterone that men are exposed to *in utero* correlates with riskier decisions in later life. Second, testosterone exposure was associated with more optimism and overweighting of small probabilities of chances under time pressure for male participants, relative to female participants.

## Motivations and hypotheses

There are two broad classes of explanation for why women are underrepresented in front office roles. The first is that women behave differently to men, and in ways which are not valued in financial services. The second group is that the front office provides an environment that neither welcomes women, nor is attractive to them. These two positions poles of the argument could be stylized as nature and nurture.

This paper focusses on the nature element of the debate. The differences between men and women begin at the moment of fertilization where the fusion of genetic material from each parent determines whether the fetus develops as a male or female. How do these biological differences play out so that, years later, men and women make, on average, very different decisions?

Biological sex is determined at conception and many of its effects are cemented *in utero*. The default pattern for developing embryos is female, but the Y chromosome contains the *SRY* gene which transforms the indifferent gonad into male testes. These testes then produce testicular hormones (e.g., testosterone) which confers the male primary and secondary sex characteristics. Between 12 and 18 weeks of gestation male fetal plasma testosterone levels reach nine times that of females causing the formation of male external genitalia and conformational alterations in the brain and spinal cord (Breedlove and Hampson, 2002). This testosterone peak also affects the length of the digits. Intra-uterine testosterone levels have been found negatively correlated with the ratio between the second and fourth digits (index and ring fingers, known as the 2D:4D ratio) (Lutchmaya et al., 2004). Higher concentrations of fetal testosterone produce lower 2D:4D ratios and men typically have lower 2D:4D ratios than women (Manning et al., 1998; McIntyre, 2006). Interestingly, no relationship between testosterone and 2D:4D ratio is observed (Hollier et al., 2015) when testosterone levels in umbilical blood are measured at birth. This may be a timing issue, as the *in utero* testosterone peak (see above) has passed and the post-partum peak (Swerdloff et al., 2002) has yet to occur.

During puberty, another androgen peak results in the development of male secondary sex characteristics and has further effects on cerebral architecture. Again, this pubertal peak affects bodily conformation, notably in the ratio between facial width and height, or fWHR (Verdonck et al., 1999; Weston et al., 2007), with males having larger ratios than females.

These markers of testosterone exposure can be readily measured and impact on risk-taking and decision-making. Coates et al. (2009) found that male traders with lower 2D:4D had higher profitability and Coates and Page (2009) found that this result was entirely driven by greater risk-taking. Garbarino et al. (2011) designed a financially motivated decision-making experiment and found that: men had lower 2D:4D ratios than women and the difference was significant; women made more risk-averse choices compared with men, and both men and women with smaller digit ratios made riskier financial choices with effect being identical for men and women. Barel (2017) found that only women exhibited more financial risk taking with lower 2D:4D but higher optimism levels. However, no significant correlation between the 2D:4D and risk preferences were observed by Schipper (2014). Drichoutis and Nayga (2015) found no effect of digit ratio on either risk or time preferences. Studies using 2D:4D ratios are potentially confounded by a number of factors such as ethnic groups (Manning et al., 2007). Consequently, the relationship between 2D:4D and risk-taking is not conclusive. Brañas-Garza et al. (2017) provide a detailed review of this research. Little is known about the associations with fWHR. The differential impact of testosterone exposure on risk preferences for both genders remains inconclusive.

The 2D:4D ratio has been shown, in men, to be negatively correlated with good visual and spatial performance (Manning and Taylor, 2001; Kempel et al., 2005), dominance and masculinity (Fink et al., 2007), sensation-seeking (Fink et al., 2006), and overconfidence (Dalton and Ghosal, 2014; Neyse et al., 2016). Overconfident investors and those investors most prone to sensation seeking were found trading more frequently (Grinblatt and Keloharju, 2009). Tester and Campbell (2007) found that the significant relationship between the 2D:4D ratio and sporting achievement was nearly identical in both men and women. However, several traits were only found in women, for instance, sensation-seeking, psychoticism, neuroticism (Austin et al., 2002), verbal fluency (Manning, 2002) social cognition (Williams et al., 2003), and cognitive reflection (Bosch-Domènech et al., 2014). The predictions of the face width-to-height ratio (fWHR) were mostly found in men. Carré and McCormick (2008) found that male undergraduate students had a larger fWHR, higher scores of trait dominance, and more reactive aggression than female students. However, the individual differences in fWHR predict reactive aggression in men but not in women. Valentine et al. (2014) supported the finding that fWHR is a physical marker of dominance and men with higher ratios are more attractive to women. Lefevre et al. (2014) suggested links between fWHR and self-reported aggression in both men and women, as well as dominance in men, but not in women.

This study examines the relation between gender and risk-taking in situations with and without time pressure. We summarize our investigation in three questions:

Question 1: Does time pressure increase an appetite for risk?
Question 2: Is higher testosterone exposure associated with higher risk-taking?
Question 3: Is there heterogeneity by gender?

## Methods

### Experimental design

The experiment was programmed in zTree (Fischbacher, 2007) and conducted at Queensland University of Technology (QUT). Participants were recruited via the Queensland Behavioral Economics Group (QuBE) website, powered by Online Recruitment System for Economics and Experiments (ORSEE) (Greiner, 2004).^1^ 154 students (74 females and 80 males) in total participated in 9 experimental sessions in this study and each experimental session lasted around 30–40 min. Upon entry to a laboratory at QuBE, participants were randomly assigned to a computer terminal. They were asked to complete the task individually and independently.

**Figure 1:**
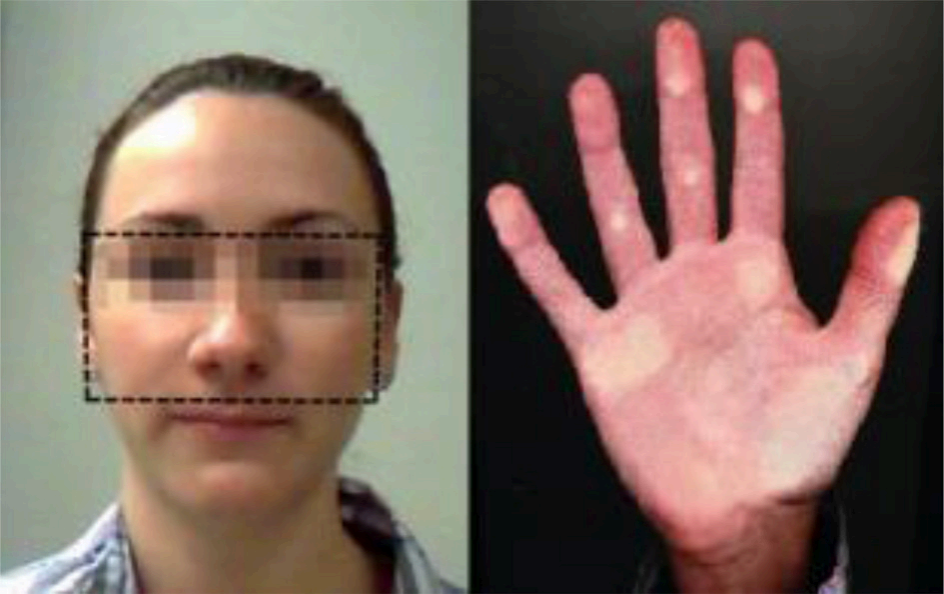
An example of the ratio measurements.

To measure the markers of participants' testosterone exposure, photographs of their faces were taken and right hands were scanned (see Figure 1). Then, the facial width was measured by the distance between the left and the right zygion (bizygomatic width) and the facial height was measured by the distance between the upper lip and brow (upper facial height Carré and McCormick, 2008, see photograph in Figure 1). The lengths of the second and fourth digits were measured from the basal crease (i.e., the crease closest to the base of the finger) to the central point of the fingertip (Garbarino et al., 2011; Neyse and Brañas-Garza, 2014).

Participants then engaged in a standard risk preference elicitation task using Random Lottery Pair design (Hey and Orme, 1994). This task consists of three phases and 30 decisions between pairs of lotteries per phase (90 decisions in total). Further, to investigate the role of time pressure, there are different time constraints imposed in each phase: no constraint, 8 and 4 s to make a decision in one lottery pair. These are the time constraints chosen by Kocher et al. (2013) in their study of risky decisions under time pressure. An 8 s constraint represents a moderate time pressure, while 4 s is a situation of extreme time pressure where participants have very little time to make a decision after discovering the different outcomes and their probability. We adopt a within-subject approach, which allows us to gain statistical power by controlling for unobservable characteristics. However, it also runs the risk of creating ordering effects. Therefore, to mitigate this risk, we randomized the order of the phases across experimental sessions.

**Figure 2:**
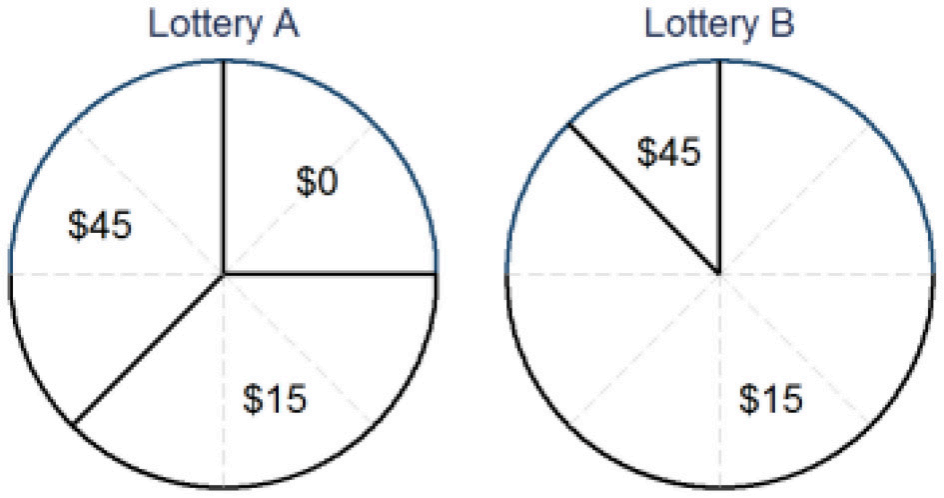
An example of lottery pairs.

Participants were presented with a pair of pie charts describing the probabilities of four fixed monetary prizes of 0, 15, 30, and $45 (Australian Dollars).^2^ An example of lottery pairs is shown in Figure 2. In this example, Lottery A offers a $0 prize with a probability of 25%, $15 with a probability of 37.5% and $45 with a probability of 37.5%, whilst Lottery B offers a $15 prize with a probability of 87.5 and $45 with a probability of 12.5%.^3^ Hence, the expected payoff is $22.5 for Lottery A and $18.75 for Lottery B. There were no numerical references to the probabilities and expected payoffs displayed; participants had to judge them from the pie chart within the given time constraint. No indifference choice was allowed between the two lotteries.

At the end of the 90 decisions, one lottery pair was randomly chosen and the participant's decision in this particular lottery pair was chosen. The “roulette wheel” of this lottery was then spun on their computer screen to determine their final payments. The instructions were explained in the form of a PowerPoint presentation before the start of the experiment, and they are shown in the Appendix.

### Estimation procedure

To study risk preferences, we fit a rank dependent utility model (RDU). We use this model because of its general form. It contains expected utility (EU) as a special case, allows us to disentangle risk preferences between a sensitivity to *payoffs* via the curvature of a *utility* function and a sensitivity to *probabilities* via the curvature of a *probability weighting* function (Wakker, 2010).

The utility of each lottery can be determined by the function:

(1) $$\begin{array}{r} V=\;\overset{K}{\underset{k=1}{\sum }}w_{k}\times U_{k} \end{array}$$

Where

$$\{\begin{array}{ll} \begin{array}{l} w_{i}=\;\omega (p_{i}+\cdots +p_{n})\\ -\omega (p_{i+1}+\cdots +p_{n}), \end{array} & i=1,\;\ldots ,\;n-1,\\ w_{i}=\;\omega (p_{i}), & \;i=n \end{array}$$

In the equations above, *k* = 1, …, *K* and *K* is the number of possible prizes in a lottery. The subscript of *w*_*i*_ indicates that the prizes were ranked from the smallest to the biggest. The probability weighting function ω(*p*) is then applied to the aggregated probabilities, so the decision weights *w*_*i*_ are derived by the differences in these transformed aggregated probabilities.

We use the power constant relative risk aversion (CRRA) utility function:

(2) $$\begin{array}{r} U\left(x\right)=\;\frac{x^{1-\alpha }}{1-\alpha } \end{array}$$

where *x* is each prize in a lottery and α (≠1) is the coefficient and yet to be estimated. If α > 0, it corresponded to a risk-averse attitude toward the actual payoff; α < 0 reflects a risk-loving attitude; α = 0 means risk-neutral.

Furthermore, we use the two-parameter weighting function in (Lattimore et al., 1992):

(3) $$\begin{array}{r} \omega \left(p\right)=\;\frac{\delta p^{\gamma }}{\delta p^{\gamma }+{\left(1-p\right)}^{\gamma }} \end{array}$$

where δ, γ > 0. The parameter γ determines the curvature (concavity or convexity) of the probability weighting. If γ > 1, the function has an “S-shape.” It means that a small probability is underweighted by the agent. For example, while in Figure 2, the probability to win $45 is 12.5% in Lottery B, an agent would act as if he/she believed this probability is lower than 12.5%. If γ < 1 the function has an “inverse S-shape.” It means that a small probability is over weighted. Then an agent thinks that his or her chance receiving $45 is >12.5%.

The parameter δ provides an additional weight on the probability weighting function. If δ < 1, the probabilities are down weighted, indicating a pessimistic view of the payoffs. For example, an agent thinks that the chance of receiving $45 is <12.5% and that the chance of receiving $15 is <87.5%. On the contrary, if δ > 1, the probabilities are over weighted, indicating that an agent holds an optimistic view toward the overall chances. Additionally, the EU is a special case when both γ = δ = 1.

We estimate these parameters using a random utility approach whereby the decision maker sometimes does not select the option with the highest utility due to cognitive errors. We use a “context utility” specification, making the variance of these cognitive errors depend on the magnitude of the payoffs being considered in the decision situation. This specification has been found to be better than alternatives which assume that errors are the same between different context of choice (Wilcox, 2011). The difference in utility between the two lotteries in a pair is modeled as:

(4) $$\begin{array}{r} \nabla V=\frac{\lambda \left(V_{A}-V_{B}\right)}{U\left(z_{max}\right)-U\left(z_{min}\right)} \end{array}$$

where λ represented the overall scale of the errors and the denominator is the influence of the specific context on the error in one lottery pair. The subscript of “A” and “B” represent the two lotteries and *z*_*max*_ and *z*_*min*_ denote the maximum and minimum possible payoffs in one pair.

The parameters α, γ, δ as the reflection of participants' risk preference, and their perception of probabilities, are estimated by maximum likelihood method by using pooled data and clustering standard errors at each participant level. Therefore, the likelihood function is written as:

(5) $$\begin{array}{l} \ln L\left(\alpha ,\gamma ,\delta ;y\right)=\sum_{m}(\left(\ln\Phi \left(\nabla V\right)|y_{m}=1\right)\\ \;+(\ln\left(1-\Phi \left(\nabla V\right)\right)|y_{m}=0)) \end{array}$$

where *y*_*m*_ = 1(0) denotes the choice of lottery A (B) chosen in each pair *m*.

For ease of interpretation by the reader (and the authors), the 2D:4D ratios were reversed as R2D:4D, so that a higher ratio suggests higher testosterone exposure—just as higher fWHR suggests higher testosterone exposure. Both ratios are standardized. We also introduce two variables: “Male” as a gender dummy variable and “Time” as a categorical variable to measure the phases under three different time constraints. The parameters α, γ and δ are written as linear combination of variables, as written by the below equations, jointly in the maximum likelihood estimation:

(6) $$\begin{array}{l} \alpha =\beta_{0}+\beta_{1}\text{Male}+\beta_{2}\text{Ratio}+\beta_{3}\text{Male}\times \text{Ratio}+\;\beta_{4}\text{Time}\\ \;+\;\beta_{5}\text{Male}\times \text{Time }+\beta_{6}\text{Ratio}\times \text{Time}\\ \;+\;\beta_{7}\text{Male}\times \text{Time}\times \text{Ratio}\\ \gamma =\mu_{0}+\mu_{1}\text{Male}+\mu_{2}\text{Ratio}+\mu_{3}\text{Male}\times \text{Ratio}+\;\mu_{4}\text{Time}\\ \;+\;\mu_{5}\text{Male}\times \text{Time }+\mu_{6}\text{Ratio}\times \text{Time}\\ \;+\;\mu_{7}\text{Male }\times \text{ Time}\times \text{Ratio}\\ \delta =\varphi_{0}+\varphi_{1}\text{Male}+\varphi_{2}\text{Ratio}+\varphi_{3}\text{Male}\times \text{Ratio}+\;\varphi_{4}\text{Time}\\ \;+\;\varphi_{5}\text{Male}\times \text{Time }+\varphi_{6}\text{Ratio}\times \text{Time}\\ \;+\;\varphi_{7}\text{Male}\times \text{Time}\times \text{Ratio}. \end{array}$$

Therefore, our estimates are the parameters leading to the highest likelihood. After estimating our structural models from (1 to 6) jointly, we obtain two sets of estimations separately by using fWHR as “Ratio” (Estimation 1 in **Table 2**) and by using R2D:4D as “Ratio” (Estimation 2 in **Table 2**) in model (6). We can then investigate the interrelation between parameters and the effects of variables, by interpreting the coefficients for the sub-groups, for example, if β_3_ is significantly not equal to 0, it means the fWHR or R2D:4D has significantly different effects on males and females' risk attitude in our experiment.

## Results

The summary statistics are presented in Table 1. The average fWHR for male participants in our experiment is 1.842 (*SD* = 0.140), and the average for females is 1.875 (*SD* = 0.108). The fWHR is not normally distributed in our sample. Therefore, we use a nonparametric test, the Mann-Whitney test to examine the differences between two gender groups. We find that male participants have lower fWHR than females (test statistic: 2.305 and *p* = 0.021). The average 2D:4D ratio for males is 0.963 (*SD* = 0.032) and for females is 0.967 (*SD* = 0.042). However, the differences in 2D:4D ratio between male and female participants in our experiment are not significant (test statistic: −1.548 and *p* = 0.122).

**Table 1:** Summary statistics.

**Subsample**	**Observations (*N*)**	**fWHR**	**2D:4D**	**Expected return of chosen lotteries**	**Variance of chosen lotteries**
Males	80	1.842 (0.140)	0.963 (0.032)	22.581 (7.46)	141.899 (120.68)
Females	74	1.875 (0.108)	0.967 (0.042)	22.579 (7.50)	134.125 (115.20)
Mann-Whitney Test (*H*_0_: Females = Males)		*z* = 2.305 *p* = 0.021	*z* = −1.548 *p* = 0.122	*z* = −0.236 *p* = 0.814	*z* = −3.263 *p* = 0.021

*Standard deviations are in the parentheses*.

The expected return of chosen lotteries for males is 22.581 (*SD* = 7.46), showing no significant difference (*p* = 0.814) from females of 22.579 (*SD* = 7.50). However, females chose the lotteries with significantly (*p* = 0.021) lower variance (134.125, *SD* = 115.20) than males (141.899, *SD* = 120.68). This suggests that female participants in our experiment have less appetite for risk. We have also used the Brown and Forsythe (1974) to examine the equality of the variances of chosen lotteries. The test result suggests that male participants have higher variances, as the Levene's robust test statistic (*W*_0_) is 10.498 with *p* = 0.001.

The CRRA function parameter α is separately estimated under EU and RDU. The EU model is simply estimated like the RDU model with the parameters γ, δ each set to 1. Results for EU and RDU parameters are presented in Table 2.

**Table 2:** Estimation Results on fWHR and R2D:4D.

**Estimation 1**	**EU**	**RDU**			**Estimation 2**	**EU**	**RDU**		
	**α**	**α**	**γ**	**δ**		**α**	**α**	**γ**	**δ**
Male (β_1_, β_1_, μ_1_, φ_1_)	−0.135^**^ (−2.37)	−0.071^***^ (−2.60)	0.059 (1.01)	0.316 (1.31)	Male (β_1_, β_1_, μ_1_, φ_1_)	−0.096^**^ (−2.12)	−0.075^**^ (−2.50)	0.054 (0.94)	0.108 (0.59)
fWHR (β_2_, β_2_, μ_2_, φ_2_)	−0.026 (−0.79)	−0.037^*^ (−1.79)	0.067 (1.32)	−0.026 (−0.14)	R2D:4D (β_2_, β_2_, μ_2_, φ_2_)	−0.021 (−0.76)	−0.037^**^ (−2.09)	0.038 (1.52)	−0.068 (−0.39)
Male × fWHR (β_3_, β_3_, μ_3_, φ_3_)	−0.115 (−1.64)	0.084^***^ (2.97)	−0.160^**^ (−2.41)	0.820^***^ (3.35)	Male × R2D:4D (β_3_, β_3_, μ_3_, φ_3_)	−0.164^**^ (−2.19)	0.069^*^ (1.80)	−0.148^**^ (−2.38)	0.945^***^ (3.24)
Under 8 s (β_4_, β_4_, μ_4_, φ_4_)	−0.183^***^ (−5.78)	−0.085^***^ (−2.82)	−0.303^***^ (−7.84)	−0.373^***^ (−3.00)	Under 8 s (β_4_, β_4_, μ_4_, φ_4_)	−0.167^***^ (−5.47)	−0.088^**^ (−2.22)	−0.297^***^ (−6.34)	−0.364^***^ (−2.90)
Under 4 s (β_4_, β_4_, μ_4_, φ_4_)	−0.045 (−1.19)	−0.020 (−0.64)	−0.157^***^ (−2.93)	−0.119 (−0.78)	Under 4 s (_β_4_, β4_, μ_4_, φ_4_)	−0.024 (−0.61)	−0.025 (−0.66)	−0.149^***^ (−2.69)	−0.168 (−1.03)
Male × Under 8 s (β_5_, β_5_, μ_5_, φ_5_)	0.017 (0.25)	−0.043 (−0.70)	−0.035 (−0.57)	−0.328 (−1.58)	Male × Under 8 s (β_5_, β_5_, μ_5_, φ_5_)	−0.014 (−0.25)	−0.010 (−0.03)	−0.031 (−0.25)	−0.112 (−0.23)
Male × Under 4 s (β_5_, β_5_, μ_5_, φ_5_)	0.007 (0.10)	0.052 (1.10)	−0.062 (−0.83)	0.049 (0.19)	Male × Under 4 s (_β_5_, β5_, μ_5_, φ_5_)	0.008 (0.13)	0.064 (1.25)	−0.064 (−0.88)	0.075 (0.32)
fWHR × Under 8 s (β_6_, β_6_, μ_6_, φ_6_)	0.084^**^ (2.22)	−0.032 (−1.17)	0.034 (0.75)	−0.051 (−0.32)	R2D:4D × Under 8 s (β_6_, β_6_, μ_6_, φ_6_)	0.064 (1.52)	−0.032 (−1.29)	0.014 (0.57)	−0.073 (−0.46)
fWHR × Under 4 s (β_6_, β_6_, μ_6_, φ_6_)	0.108^***^ (2.78)	−0.008 (−0.25)	0.052 (0.72)	−0.276 (−1.59)	R2D:4D × Under 4 s (_β_6_, β6_, μ_6_, φ_6_)	0.058^*^ (1.71)	−0.003 (−0.06)	0.042 (1.23)	−0.147 (−0.48)
Male × fWHR × Under 8 s (β_7_, β_7_, μ_7_, φ_7_)	−0.158^*^ (−1.76)	−0.067 (−0.97)	−0.032 (−0.46)	−0.650^***^ (−2.97)	Male × R2D:4D × Under 8 s (β_7_, β_7_, μ_7_, φ_7_)	−0.288^***^ (−2.68)	−0.097 (−0.11)	−0.031 (−0.07)	−0.558 (−0.59)
Male × fWHR × Under 4 s (_β_7_, β7_, μ_7_, φ_7_)	−0.162^**^ (−1.99)	0.057 (1.49)	−0.027 (−0.32)	0.378^*^ (1.69)	Male × R2D:4D × Under 4 s (β_7_, β_7_, μ_7_, φ_7_)	−0.169 (−1.37)	0.029 (0.44)	−0.028 (−0.44)	−0.011 (−0.03)
Constant (β_0_, β_0_, μ_0_, φ_0_)	0.480^***^ (15.43)	0.558^***^ (33.68)	0.844^***^ (20.54)	1.394^***^ (10.31)	Constant (β_0_, β_0_, μ_0_, φ_0_)	0.476^***^ (15.70)	0.549^***^ (34.80)	0.853^***^ (18.18)	1.381^***^ (11.19)

*Z statistics in parentheses*,

* *p < 0.1*,

** *p < 0.05*,

*** *p < 0.01*.

We find that participants tend to have a concave utility function reflecting risk aversion (α > 0), both in the EU and RDEU estimation. We also find that the probability weighting function displays the typical “inverse S-shape” with the parameter γ being below 1 for men and women. These results are consistent with previous findings (Harrison and Rutström, 2008; Bruhin et al., 2010).

Q3: We find that males are less risk averse both in the EU and RDU estimations (β_1_ < 0 in Estimation 1 and 2). However, we do not find baseline gender differences in probability perception (the coefficients μ_1_ and φ_1_ are not significantly different from 0 in Table 2).^4^

Q2 and Q3: There is some indication of a link between exposure and risk aversion. We find that R2D:4D has a negative effect on the risk-attitude parameter α only for males, but not for females (β_3_ in Estimation 2 is −0.164 and significant with *p* < 0.05) in the EU estimations. It shows that males with higher R2D:4D have more appetite for risk (less risk-averse). We do not find an association between fWHR and any changes of risk taking^5^ in the EU estimations (β_2_ in Estimation 1 and 2 are not significant).

In the RDU estimations, we find that fWHR (β_2_ is −0.037 with *p* < 0.1 in Estimation 1) and R2D:4D (β_2_ is −0.037 with *p* < 0.05 in Estimation 2) have a negative effect on females' risk-attitude, but positive effect on males' risk-attitude (β_3_ is 0.084 with *p* < 0.01 and 0.069 with *p* < 0.1 in Estimation 1 and 2). This suggests that females with higher ratios have more appetite for risk (less risk-averse), while the relationship is opposite for the males.

The differences in α across the two models are to be expected. The reason is that the risk attitudes are only represented by α in the EU model, while they are represented by α, γ, and δ in the RDU model. In the case where EU is the best model, we should expect the RDU model to have a similar α and γ = 1, δ = 1. Whenever people weight probabilities, γ and δ are going to differ from 1. In such a case, there is no reason to expect the α to be the same in the EU and RDU as the α in the EU will partially adjust itself to explain part of the risk attitudes reflected in the γ and δ in the RDEU model.

There is a clearer indication of a link with the attitudes to probabilities for males (but not for female participants). The inverse S-shape of the probability weighting function is more pronounced for males with higher ratios (μ_3_ is −0.160, *p* < 0.05 in Estimation 1 and −0.148, *p* < 0.05 in Estimation 2). And male participants with higher ratios are more optimistic (φ_3_ is 0.820, *p* < 0.01 in Estimation 1 and 0.945, *p* < 0.01 in Estimation 2). It suggests that male participants with higher ratios overweight their chances of receiving bigger payoffs and are more optimistic toward their chances of winning monetary outcomes. A similar association was not found for female participants.

Q1: We find some indication that time pressure increases risk aversion with α being smaller in the 8 s time pressure condition (β_4_ is −0.183 with *p* < 0.01 and −0.167 with *p* < 0.01 in Estimation 1 and 2). This result is in line with previous findings (Kocher and Sutter, 2006). However, we do not find an overall significant effect in our extreme time pressure condition (4 s).

There is also a clear effect of time pressure on the probability weighting parameter. The “inverse S-shape” appears more pronounced in the time pressure conditions (μ_4_ is −0.303 with *p* < 0.01 for 8 s and −0.157 with *p* < 0.01 for 4 s in Estimation 1; μ_4_ is −0.297 with *p* < 0.01 for 8 s and −0.149 with *p* < 0.01 for 4 s in Estimation 2). We also find more optimism, but only in the 8 s time pressure condition (φ_4_ is −0.373 with *p* < 0.01 and −0.364 with *p* < 0.01 for 8 s in Estimations 1 and 2).

Q1 and Q3: However, we do not find notable baseline gender differences in risk attitude under time pressure (8 and 4 s conditions), as β_5_, μ_5_ and φ_5_ are not significantly different from zero in Estimation 1 and 2.^6^

Q1, Q2, and Q3: When looking at the coefficient of risk aversion, there is a differential effect of time pressure by gender as a function of fWHR in the 8 s time pressure condition (β_6_ is 0.084 with *p* < 0.05 while β_7_ is −0.158 and marginally significant with *p* < 0.1 in Estimation 1). In the phase with extreme time pressure, we also find that female participants with higher fWHR have more risk-averse attitude, while males with higher ratios have more appetite for risk (β_6_ is 0.108 with *p* < 0.01 while β_7_ is −0.162 with *p* < 0.05 in Estimation 1). Further, in the Estimation 2, we find that female participants with higher R2D:4D have more risk-averse attitude in the 4 s time pressure condition (β_6_ is 0.058 and marginally significant with *p* < 0.1), whereas males with higher R2D:4D have more appetite for risk in the 8 s time pressure condition (β_7_ is −0.288 with *p* < 0.01).

**Figure 3:**
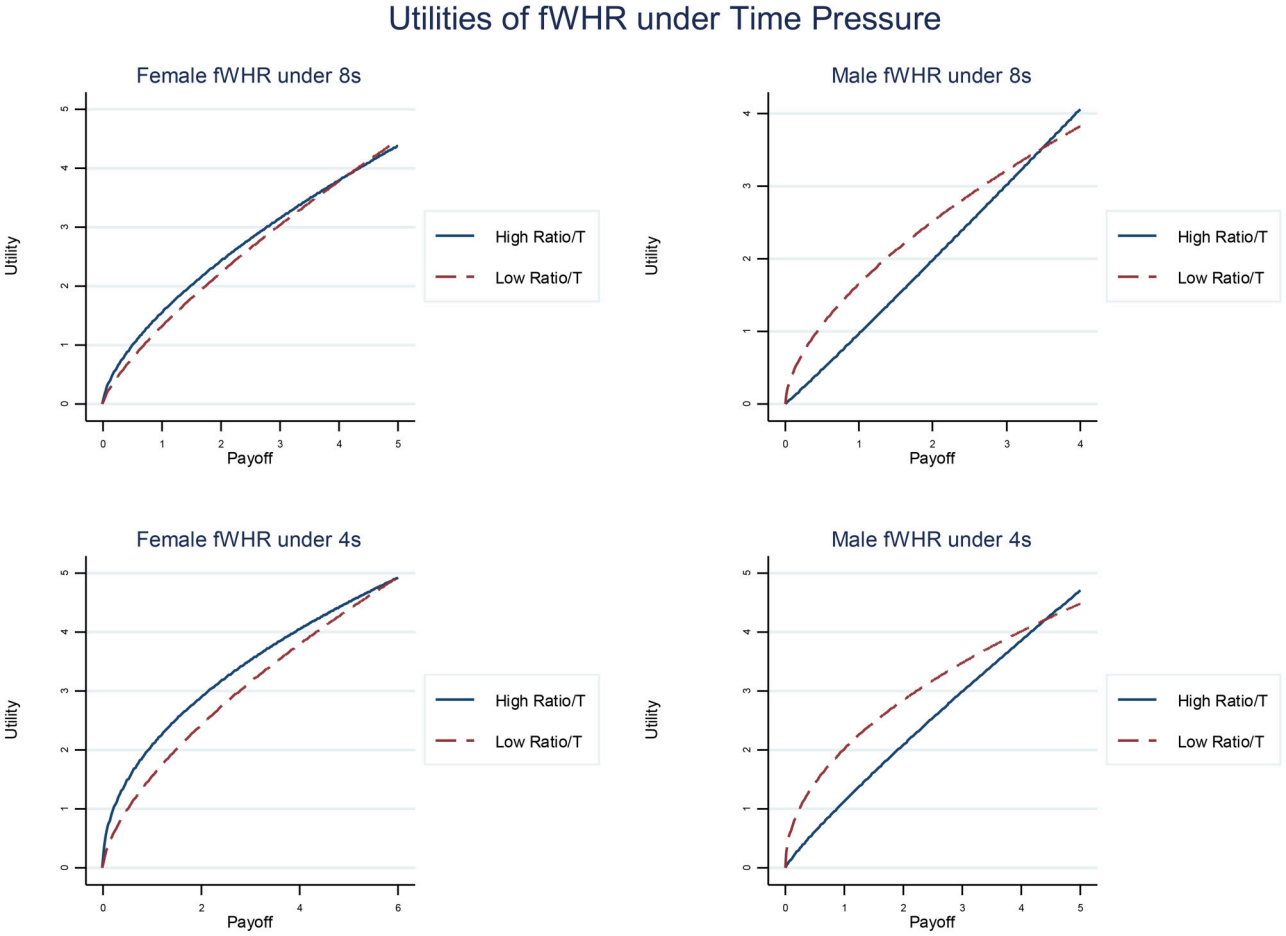
Estimated utility functions of fWHR separated by male and female sub-group and 8 and 4 s time phases. Male participants with high ratio become visibly less risk averse (the curve concavity indicates risk aversion).^7^

**Figure 4:**
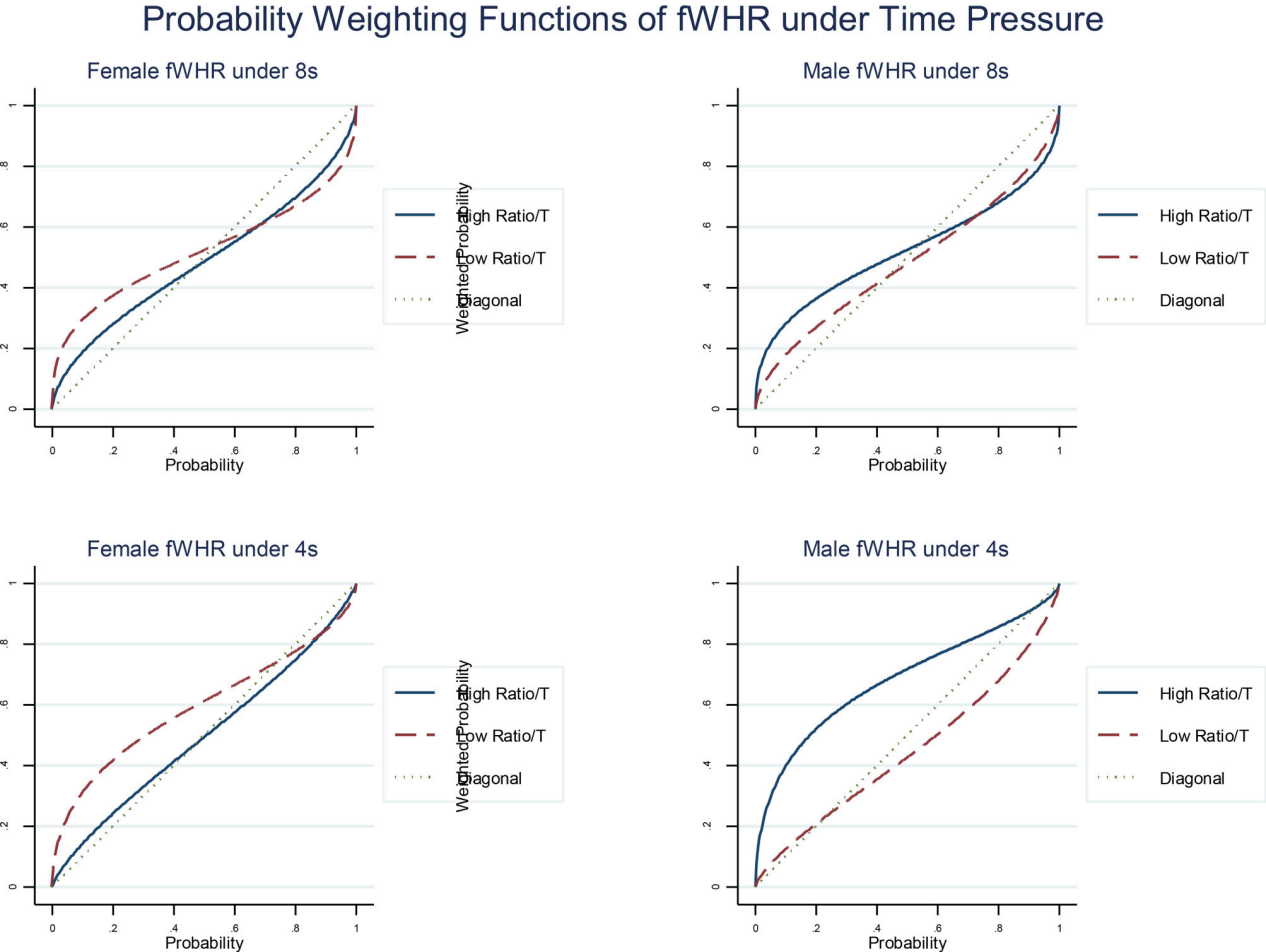
Estimated probability weighting functions of fWHR separated by male and female sub-group and 8 and 4 s time phases. The diagonal presents the actual probabilities shown in the lottery pairs in our experiment.^8^

The previous results decompose the effect on risk attitudes and probability perception of gender, prenatal exposure and time pressure. Once this decomposition is done, we can look into how different subgroups differ. We present here our estimation of the parameters α (see Figure 3), γ and δ (see Figure 4) at the aggregated level for meaningful subgroups for the fWHR ratios (overall, similar results are found for 2D:4D).

In terms of sensitivity to outcomes, the male subgroup with higher fWHR shows less curvature under time pressure in their utility function than that with lower ratios (see right column in Figure 3), but similar association is not found in female subgroup (see left column in Figure 3). The curvature in utility suggests that the risk attitude of an agent: concave as risk-averse α > 0; convex as risk-loving α < 0. Less curvature in utility function suggests more appetite for risk.

As the prizes in the lottery are rearranged from the biggest to the smallest in a rank-dependent manner, the left bottom region in a probability weighting function reveals if the probabilities of the prizes are over weighted or under weighted. For example, in any subfigure in Figure 4, the estimated functions are above the diagonal line in the left bottom region. It means that the actual probabilities are over weighted.

In terms of sensitivity to probability, under time pressure, males, with higher fWHR overestimate probabilities (Figure 4) of receiving bigger payoffs and have a more optimistic view about probabilities than those with lower ratios. However, we observe the opposite effect in the female sub-group (see left column in Figure 4). These effects are more pronounced under extreme time pressure (by comparing the top and bottom rows in Figure 4).

To answer our Questions 1–3 in section Motivations and Hypothesesdirectly, the equations (6) in our structural models are also estimated by using: (1) firstly, the “Time” variable, which is a categorical variable to measure the phases under three different time constraints, as covariates; (2) then adding “Male” and “Ratio” variables into the covariates; (3) finally, adding the interactions in to the covariates. The EU estimates are shown in Table 3 in Appendix (Supplementary Material) and the RDU estimates are shown in Tables 4, 5 in Appendix (Supplementary Material).

Based on our findings discussed above, we can now answer the three questions raised in section Motivations and Hypotheses and summarize our results:

Result 1: Time pressure increases an appetite for risk. Participants under time pressure become more optimistic.
Result 2: We do not find enough evidence to support the hypothesis that higher testosterone exposure is associated with higher risk-taking. We observed mixed results in EU and RDU estimates.
Result 3: We find that male participants are less risk averse and that the gender gap in risk attitudes increases under moderate time pressure. We also observe that men with higher testosterone exposure exhibit a greater optimism and overestimation of small probabilities of success.

## Discussion and conclusion

This study looked into gender difference in risk attitude under pressure and the potential role of prenatal exposure. We find that males are less risk averse than female participants, in line with existing research. We disentangled the different aspects of risk preferences, giving us new insights into these gender differences. We found that gender differences were clearer in the sensitivity to probability than in the sensitivity to outcomes.

When looking at prenatal exposure to testosterone, we find that males with high fWHR and R2D:4D sought more risk and overweighted small probabilities of high gain. They also were more optimistic about outcomes than the females. Females with high fWHR and R2D:4D did the opposite, taking less risk. Time pressure also, on average, made males more optimistic.

In summary, men, and particularly those with high fWHR and R2D:4D took more risk and were more bullish about pursuing an elusive chance of winning, especially under time pressure.

These results show that prenatal testosterone exposure alters risk-taking in men; particularly under time pressure. Previous research has shown that a low 2D:4D (or high R2D:4D) ratio associated with high testosterone exposure predicted a longer survival of professional traders (Coates and Page, 2009). As a consequence, men with a low 2D:4D ratio were likely to be overrepresented in the population of traders. Our result may help make sense of this finding given that the male participants with low 2D:4D ratios displayed a greater propensity to take risks under time pressure. The results of the present research did not find such an effect of time pressure on women. If women traders are seen as taking fewer risks than their male counterparts, particularly in response to time pressure, then, in a market which values activity, they may be seen as less appropriate candidates. Moreover, if they make it past the selection phase, they may well not be retained as they do not measure up to the accepted yardstick for performance.

As well as demonstrating marked differences between men and women in decision making, this research also clearly confirms that preferences are not stable and that time pressure affects choice. Because each participant was exposed to the same information in each case, there was no information difference. Rather time pressure was likely to have, interfered with information processing, thereby producing differing results. The nature of this instability was complex, being influenced by both time pressure and the long-term organizational effects of testosterone. Previous experimental and theoretical studies have argued that deliberation may have a non-linear effect on moral choices (Moore and Tenbrunsel, 2014) and cooperation (Capraro and Cococcioni, 2016). A non-linear relationship has also be observed by some authors between circulating testosterone and risk taking (Stanton et al., 2011). The consequence of all this is that the useful simplification of assuming that preferences are stable, may lead to forget the fact that preference instability is substantial, widespread and non-linear.

Our results suggest that if the market privileges risk taking and confidence under time pressure then a combination of physiological predisposition and preference instability may favor the employment of men. This, in turn, may explain the preponderance of men in the market. This is difficult to prove in any definitive sense as counterfactuals are not readily available. Care also has to be taken in extrapolating from a laboratory study to global markets as the requirements of controlling for factors except those under investigation inevitably means that a degree of verisimilitude is lost. The risk-taking task, for example is a stylized one with a limited number of parameters. The sample size, relative to financial markets, is small, and does not, necessarily, mirror the profile of those in financial markets. Moreover, the choices are single shot interactions, rather than the dynamic, ongoing and varied interactions observed in real markets. This study only looks at the organizational effects of testosterone manifest in 2D:4D ratio and fWHR, not at the activational effects of circulating testosterone. It also does not address other hormones, such as cortisol, which have been demonstrated to affect risk-taking (Kandasamy et al., 2014). Despite this, our findings on gender differences, the role of prenatal testosterone exposure and of time pressure provide some clues as to why women may be at a perceived disadvantage in a pressurized trading environment. This, in turn, may mean that they are less likely to be recruited and retained.

To provide a fuller picture, there are a number of questions for further research to address. The first is to examine risk taking when the probability distribution is less clearly defined. This ambiguity may affect the results. The second question is whether the nature of risk taking changes when there is interaction between participants. These sorts of interaction studies have been undertaken in hormone research (e.g., Cueva et al., 2015). They improve external validity but sometimes at the expense of mechanistic clarity. Third, external validity could be improved by conducting the task with different groups of bank employees. It may be that different functions have different risk profiles, so traders may differ from asset managers, for example. Fourth, further research should sample circulating hormone levels to explore the interaction between activational (circulating) and organizational (i.e., those shaping development) hormones.

This research provides a piece of the puzzle as to why women are underrepresented in a number of areas of finance. But does it matter that these areas are male dominated? Markets are well-served by diversity as a means of tempering herd instincts. A market that is skewed in favor of employing men may, therefore, bring its own set of problems. Some researchers, for example, Coates and colleagues (Coates et al., 2010), have suggested that improving gender diversity may improve market stability. This is supported by experimental evidence which suggested both gender (Cueva and Rustichini, 2015) and hormonal diversity (Cueva et al., 2015) improve market stability. Although there are many explanations for aggregate behavior in financial markets, the effect of gender, preference stability and hormonal exposure may have significant repercussions.

## Ethics statement

This study was carried out in accordance with the recommendations of the ethics committee from School of Economics and Finance at Queensland University of Technology with written informed consent from all subjects. All subjects gave written informed consent in accordance with the Declaration of Helsinki. The protocol was approved by the ethics committee from School of Economics and Finance at Queensland University of Technology.

## Author contributions

All authors listed have made a substantial, direct and intellectual contribution to the work, and approved it for publication.

### Conflict of interest statement

The authors declare that the research was conducted in the absence of any commercial or financial relationships that could be construed as a potential conflict of interest.

## Footnotes

^1^The research ethics require participants being anonymous and unidentifiable during and after the experiment, therefore participants' personal information, such as age, faculty and ethnic groups were not collected. The ethics committee at QUT Business School approved this research and participants gave written informed consents before partaking the experiment.

^2^We used the set of lotteries from Conte et al. (2011) and Hey (2001) also used by many other studies including Moffatt (2005) and Conte et al. (2011). Hey (2001) explained the logic of the choice of lotteries. Each lottery can be associated with a point in the Marschak-Machina triangle (space representing all possible lotteries with three outcomes). In this triangle, EU decision makers have linear indifference curves. The selection of lotteries creates pairs of points in the triangle, by varying the location of these pairs of points, the choice among lotteries reveals the slopes of the indifference curves in the triangle and whether these slopes are not constant (revealing that decision makers violate EU, for instance because they weight probabilities).

^3^The lotteries used in each phase had different probabilities, however, they were drawn randomly from the same pool. They did not differ in characteristics on average. The number of lotteries in each phase (30 decisions) limits the risk of substantial differences across phase due to the random selection of lotteries.

^4^Note that gender differences can still exist overall due to gender differences in other variables such as prenatal exposure to testosterone which can have an influence on risk preferences. We look into this below.

^5^The β_3_ in Estimation 1 is −0.115, but not significant as *p* > 0.1 in the EU estimations.

^6^Gender differences in risk attitude under time pressure can still be present due to gender differences in prenatal exposure to testosterone. We look into this below.

^7^We calculate the utilities based on our estimations of Estimation 1 and 2 in Table 2. As fWHR and R2D:4D are standardized, we use value of 1 as high ratio and −1 as low ratio. Therefore, for example, the utility for females with high ratios in the 8s time condition is calculated as: α = 0.480−0.026 × 1−0.183+0.084 × 1 = 0.355, and the utility for low ratios is 0.239. The utilities for males in the 8s time condition are calculated as −0.036 for high ratios and 0.395 for low ratios. The utilities for females in the 4s time condition are calculated are 0.517 for high ratios and 0.354 for low ratios, and that for males are 0.112 for high ratios and 0.503 for low ratios.

^8^The calculations of the probability weighting functions apply the same method of calculations in the utilities in Figure 3.
