# Supplementary material for: Investigating Gender Differences under Time Pressure in Financial Risk Taking
Source: Front Behav Neurosci. 2017 Dec 15;11:246. doi: 10.3389/fnbeh.2017.00246 (PMC5736567; doi:10.3389/fnbeh.2017.00246)
Supplement: Supplementary file 1 [file DataSheet1.docx]

# Appendix

The instructions in the experiment discussed in Section 3.1 were presented by the following slides in PowerPoint:

| 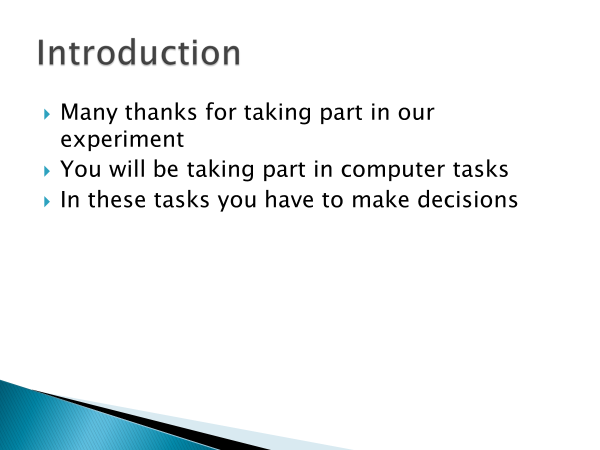 | 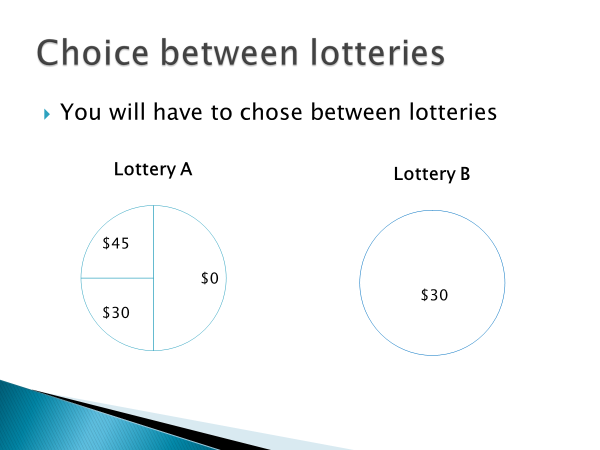 |
| --- | --- |
| (1) | (2) |
| 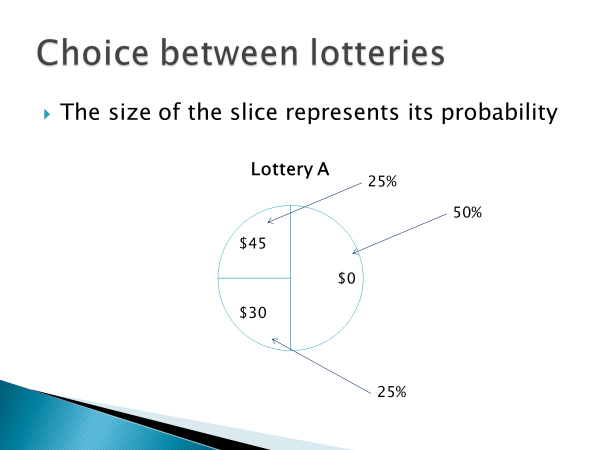 | 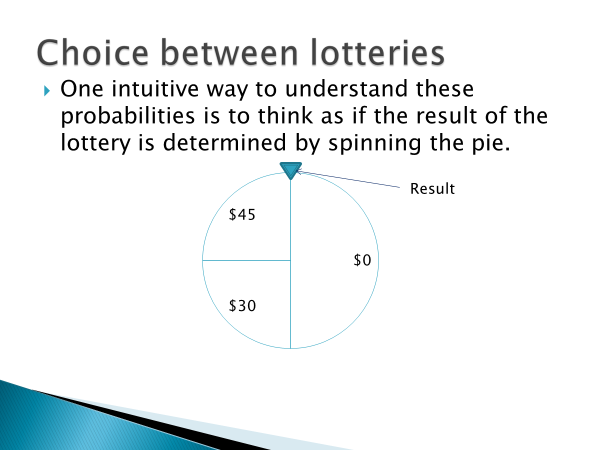 |
| (3) | (4) |
|  |  |
| 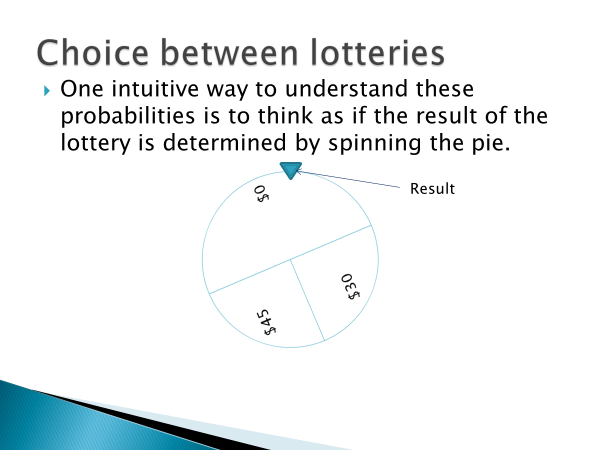 | 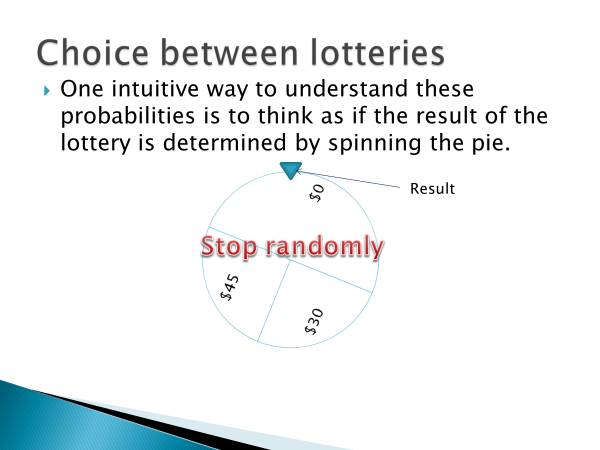 |
| (5) | (6) |
| 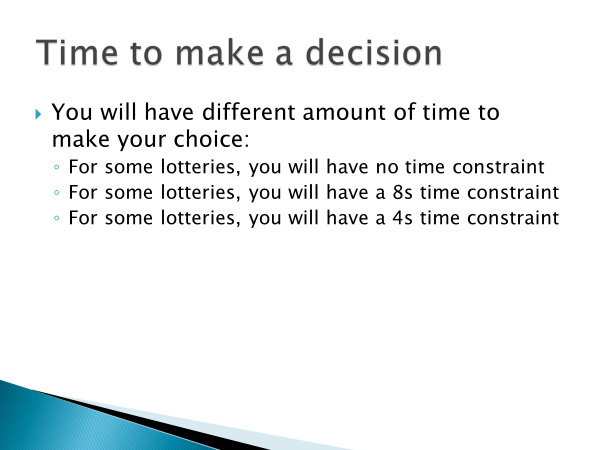 | 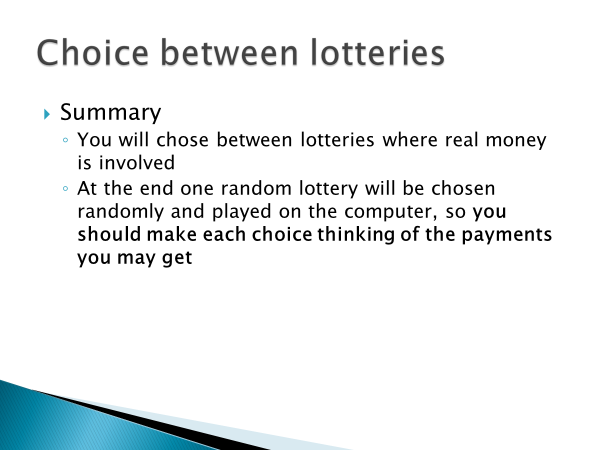 |
| (7) | (8) |

| Table 3: EU Estimation Results on fWHR and R2D:4D | | | | | | | |
| --- | --- | --- | --- | --- | --- | --- | --- |
| Estimation 1 | EU | | | Estimation 2 | EU | | |
|  | $\alpha$ | $\alpha$ | $\alpha$ |  | $\alpha$ | $\alpha$ | $\alpha$ |
| Male  ($\beta_{1}$) | ---- | -0.093**  (-2.03) | -0.135**  (-2.37) | Male  ($\beta_{1}$) | ---- | -0.063  (-1.50) | -0.096**  (-2.12) |
| fWHR  ($\beta_{2}$) | ---- | -0.067**  (-2.18) | -0.026  (-0.79) | R2D:4D  ($\beta_{2}$) | ---- | -0.048**  (-2.11) | -0.021  (-0.76) |
| Male$\times$fWHR  ($\beta_{3})$ | ---- | ---- | -0.115  (-1.64) | Male$\times$ R2D:4D  ($\beta_{3}$) | ---- | ---- | -0.164**  (-2.19) |
| Under 8s  ($\beta_{4})$ | -0.143***  (-6.29) | -0.146***  (-7.09) | -0.183***  (-5.78) | Under 8s  ($\beta_{4})$ | -0.143***  (-6.29) | -0.145***  (-6.89) | -0.167***  (-5.47) |
| Under 4s  ($\beta_{4}$) | -0.020  (-0.64) | -0.20  (-0.66) | -0.045  (-1.19) | Under 4s  ($\beta_{4}$) | -0.020  (-0.64) | -0.018***  (-0.57) | -0.024  (-0.61) |
| Male$\times$Under 8s  ($\beta_{5}$) | ---- | ---- | 0.017  (0.25) | Male$\times$Under 8s  ($\beta_{5}$) | ---- | ---- | -0.014  (-0.25) |
| Male$\times$Under 4s  ($\beta_{5}$) | ---- | ---- | 0.007  (0.10) | Male$\times$Under 4s  ($\beta_{5}$) | ---- | ---- | 0.008  (0.13) |
| fWHR$\times$Under 8s  ($\beta_{6}$) | ---- | ---- | 0.084**  (2.22) | R2D:4D$\times$Under 8s  ($\beta_{6}$) | ---- | ---- | 0.064  (1.52) |
| fWHR$\times$Under 4s  ($\beta_{6}$) | ---- | ---- | 0.108***  (2.78) | R2D:4D$\times$Under 4s  ($\beta_{6}$) | ---- | ---- | 0.058*  (1.71) |
| Male$\times$fWHR$\times$Under 8s  ($\beta_{7}$) | ---- | ---- | -0.158*  (-1.76) | Male$\times R2D:4D \times$Under 8s  ($\beta_{7}$) | ---- | ---- | -0.288***  (-2.68) |
| Male$\times$fWHR$\times$Under 4s  ($\beta_{7}$) | ---- | ---- | -0.162**  (-1.99) | Male$\times R2D:4D \times$Under 4s  ($\beta_{7}$) | ---- | ---- | -0.169  ( -1.37) |
| Constant  ($\beta_{0}$) | 0.459***  (16.44) | 0.844***  (20.54) | 0.480***  (15.43) | Constant  ($\beta_{0}$) | 0.459***  (16.44) | 0.853***  (18.18) | 0.476***  (15.70) |
| *z* statistics in parentheses, ^*^ *p* < 0.1, ^**^ *p* < 0.05, ^***^ *p* < 0.01. | | | | | | | |

| Table 4: RDU Estimation Results on fWHR | | | | | | | | | |
| --- | --- | --- | --- | --- | --- | --- | --- | --- | --- |
|  | $\alpha$ | $\gamma$ | $\delta$ | $\alpha$ | $\gamma$ | $\delta$ | $\alpha$ | $\gamma$ | $\delta$ |
| Male  ($\beta_{1},\mu_{1},\varphi_{1}$) | ---- | ---- | ---- | -0.080  (-1.05) | 0.013  (0.26) | 0.023  (0.14) | -0.071***  (-2.60) | 0.059  (1.01) | 0.316  (1.31) |
| fWHR  ($\beta_{2},\mu_{2},\varphi_{2}$) | ---- | ---- | ---- | -0.020  (-0.36) | -0.009  (-0.30) | 0.138  (0.96) | -0.037*  (-1.79) | 0.067  (1.32) | -0.026  (-0.14) |
| Male$\times$fWHR  ($\beta_{3},\mu_{3},\varphi_{3}$) | ---- | ---- | ---- | ---- | ---- | ---- | 0.084***  (2.97) | -0.160**  (-2.41) | 0.820***  (3.35) |
| Under 8s  ($\beta_{4},\mu_{4},\varphi_{4}$) | -0.079***  (-2.72) | -0.302***  (-9.99) | -0.335***  (-3.94) | -0.078***  (-3.17) | -0.307***  (-10.55) | -0.308***  (-3.64) | -0.085***  (-2.82) | -0.303***  (-7.84) | -0.373***  (-3.00) |
| Under 4s  ($\beta_{4},\mu_{4},\varphi_{4}$) | 0.004  0.15) | -0.175***  (-4.84) | -0.112  (-1.09) | -0.006  (-0.14) | -0.182***  (-4.62) | -0.123  (-1.02) | -0.020  (-0.64) | -0.157***  (-2.93) | -0.119  (-0.78) |
| Male$\times$Under 8s  ($\beta_{5},\mu_{5},\varphi_{5}$) | ---- | ---- | ---- | ---- | ---- | ---- | -0.043  (-0.70) | -0.035  (-0.57) | -0.328  (-1.58) |
| Male$\times$Under 4s  ($\beta_{5},\mu_{5},\varphi_{5}$) | ---- | ---- | ---- | ---- | ---- | ---- | 0.052  (1.10) | -0.062  (-0.83) | 0.049  (0.19) |
| fWHR$\times$Under 8s  ($\beta_{6},\mu_{6},\varphi_{6}$) | ---- | ---- | ---- | ---- | ---- | ---- | -0.032  (-1.17) | 0.034  (0.75) | -0.051  (-0.32) |
| fWHR$\times$Under 4s  ($\beta_{6},\mu_{6},\varphi_{6}$) | ---- | ---- | ---- | ---- | ---- | ---- | -0.008  (-0.25) | 0.052  (0.72) | -0.276  (-1.59) |
| Male$\times$fWHR$\times$Under 8s  ($\beta_{7},\mu_{7},\varphi_{7}$) | ---- | ---- | ---- | ---- | ---- | ---- | -0.067  (-0.97) | -0.032  (-0.46) | -0.650***  (-2.97) |
| Male$\times$fWHR$\times$Under 4s  ($\beta_{7},\mu_{7},\varphi_{7}$) | ---- | ---- | ---- | ---- | ---- | ---- | 0.057  (1.49) | -0.027  (-0.32) | 0.378*  (1.69) |
| Constant  ($\beta_{0},\mu_{0},\varphi_{0}$) | 0.512***  (29.10) | 0.859***  (30.20) | 1.344***  (14.46) | 0.540***  (17.17) | 0.860***  (21.24) | 1.313***  (11.34) | 0.558***  (33.68) | 0.844***  (20.54) | 1.394***  (10.31) |
| *z* statistics in parentheses, ^*^ *p* < 0.1, ^**^ *p* < 0.05, ^***^ *p* < 0.01. | | | | | | | | | |

| Table 5: RDU Estimation Results on R2D:4D | | | | | | | | | |
| --- | --- | --- | --- | --- | --- | --- | --- | --- | --- |
|  | $\alpha$ | $\gamma$ | $\delta$ | $\alpha$ | $\gamma$ | $\delta$ | $\alpha$ | $\gamma$ | $\delta$ |
| Male  ($\beta_{1},\mu_{1},\varphi_{1}$) | ---- | ---- | ---- | -0.086**  (-2.43) | 0.021  (0.55) | -0.057  (0.45) | -0.075**  (-2.50) | 0.054  (0.94) | 0.108  (0.59) |
| R2D:4D  ($\beta_{2},\mu_{2},\varphi_{2}$) | ---- | ---- | ---- | -0.035*  (-1.86) | 0.013  (0.60) | 0.077  (1.13) | -0.037**  (-2.09) | 0.038  (1.52) | -0.068  (-0.39) |
| Male$\times$ R2D:4D  ($\beta_{3},\mu_{3},\varphi_{3}$) | ---- | ---- | ---- | ---- | ---- | ---- | 0.069*  (1.80) | -0.148**  (-2.38) | 0.945***  (3.24) |
| Under 8s  ($\beta_{4},\mu_{4},\varphi_{4}$) | -0.079***  (-2.72) | -0.302***  (-9.99) | -0.335***  (-3.94) | -0.075***  (-3.36) | -0.306***  (-10.71) | -0.313***  (-3.90) | -0.088**  (-2.22) | -0.297***  (-6.34) | -0.364***  (-2.90) |
| Under 4s  ($\beta_{4},\mu_{4},\varphi_{4}$) | 0.004  0.15) | -0.175***  (-4.84) | -0.112  (-1.09) | -0.018  (-0.59) | -0.175***  (-4.56) | -0.152  (-1.47) | -0.025  (-0.66) | -0.149***  (-2.69) | -0.168  (-1.03) |
| Male$\times$Under 8s  ($\beta_{5},\mu_{5},\varphi_{5}$) | ---- | ---- | ---- | ---- | ---- | ---- | -0.010  (-0.03) | -0.031  (-0.25) | -0.112  (-0.23) |
| Male$\times$Under 4s  ($\beta_{5},\mu_{5},\varphi_{5}$) | ---- | ---- | ---- | ---- | ---- | ---- | 0.064  (1.25) | -0.064  (-0.88) | 0.075  (0.32) |
| R2D:4D$\times$Under 8s  ($\beta_{6},\mu_{6},\varphi_{6}$) | ---- | ---- | ---- | ---- | ---- | ---- | -0.032  (-1.29) | 0.014  (0.57) | -0.073  ( -0.46) |
| R2D:4D$\times$Under 4s  ($\beta_{6},\mu_{6},\varphi_{6}$) | ---- | ---- | ---- | ---- | ---- | ---- | -0.003  (-0.06) | 0.042  (1.23) | -0.147  (-0.48) |
| Male$\times$fWHR$\times$Under 8s  ($\beta_{7},\mu_{7},\varphi_{7}$) | ---- | ---- | ---- | ---- | ---- | ---- | -0.097  ( -0.11) | -0.031  (-0.07) | -0.558  (-0.59) |
| Male$\times$fWHR$\times$Under 4s  ($\beta_{7},\mu_{7},\varphi_{7}$) | ---- | ---- | ---- | ---- | ---- | ---- | 0.029  (0.44) | -0.028  (-0.44) | -0.011  (-0.03) |
| Constant  ($\beta_{0},\mu_{0},\varphi_{0}$) | 0.512***  (29.10) | 0.859***  (30.20) | 1.344***  (14.46) | 0.541***  (29.65) | 0.853***  (25.60) | 1.352***  (13.22) | 0.549***  (34.80) | 0.853***  (18.18) | 1.381***  (11.19) |
| *z* statistics in parentheses, ^*^ *p* < 0.1, ^**^ *p* < 0.05, ^***^ *p* < 0.01. | | | | | | | | | |
